# Lipid peroxidation diminishing perspective of isolated theaflavins and thearubigins from black tea in arginine induced renal malfunctional rats

**DOI:** 10.1186/s12944-018-0808-3

**Published:** 2018-07-18

**Authors:** Ali Imran, Muhammad Umair Arshad, Muhammad Sajid Arshad, Muhammad Imran, Farhan Saeed, Muhammad Sohaib

**Affiliations:** 1 Institute of Home and Food Sciences, Government College University, Faisalabad, 38040 Pakistan; 2 Department of Food Science and Human Nutrition, University of Veterinary and Animal Sciences, Lahore, 54000 Pakistan; 3 University Institute of Diet and Nutritional Sciences, Faculty of Allied Health Sciences, The University Of Lahore-Pakistan, Lahore, Pakistan

## Abstract

**Background:**

Recently oxidative stress induced maladies have amplified owing to sedentary lifestyle and monotonous diet. Introduction of plant based biomolecules may be a suitable strategy to cope with the lipid peroxidation. In this context, black tea polyphenols (theaflavin & thearubigins) are in fame among the scientific community as cost effective therapeutic agents owing to their safety, economics, structural diversity and ability to modulate various lipid peroxidation responses by halting the expression of different metabolic targets.

**Methods:**

The mandate of present investigation was to first time check the synergism among the isolated theaflavins & thearubigins against lipid peroxidative indicators both in vitro and in vivo. Purposely, theaflavins and thearubigins were isolated from black tea through solvent partition methods by using different solvents (Aqueous ethanol, Aqueous methanol & Water) and time intervals (30, 60 & 90 min) and subjected to in vitro characterization through different antioxidant indices to access the in vitro lipid peroxidation shooting effect of these bioactive moieties. Moreover, individual theaflavins contents also estimate through HPLC. For evaluation of in vivo antioxidant effect, renal malfunction was induced through arginine and forty rats were divided in four groups (10 each after power analysis) and 04 types of diets were given i.e. T_0_ (control diet without supplementation), T1 (Basic experimental Diet+ theaflavins supplementation @ 1 g), T2 (Basic experimental Diet+ Thearubigins supplementation @ 1 g) & T3 (Basic experimental Diet+ Supplementation of theaflavins+ thearubigins @ 0.5 + 0.5 g, respectively) for the period of 56 days. Alongside, a control study was also carried out for comparison by involving normal rats fed on arginine free diet. The body weight, lipid profile, glycemic responses, Renal function test, liver function test, antioxidant indices and hematological parameters were estimated at the termination of study.

**Results:**

The results indicated that theaflavins and thearubigins isolation was significantly affected by time of extraction and solvent. In this context, aqueous ethanol at 60 min extraction interval caused maximum extraction. Likewise, theaflavins isolate exhibited more antioxidant activity as compared to thearubigins. Moreover, the theaflavins and thearubigins based experimental diets imparted significant reduction in Lipid profile, glucose content, renal function tests and TBARS with enhancement in insulin, HDL and hematological parameters. In this context, theaflavin based diet caused maximum reduction in lipid profile and TBARS better as compared to thearubigins and theaflavins + thearubigins based. However, theaflavin+ thearubigins based diet caused highest glucose, urea & creatinine decline and maximum insulin increase & antioxidant indices as compared to other nutraceuticals.

**Conclusions:**

It was deduced that theaflavins & thearubigins have strong antioxidative potential both in in vitro as well as in vivo to tackle the menace associated with lipid peroxidation.

## Background

Globally, therapeutic worth of tea has been established against numerous maladies. The health escalating perspectives of tea has been attributed to his distinguished polyphenols. Accordingly, theaflavins and thearubigins are the promising polyphenols belong to subclass flavanolos. They produced when fresh tea leaves undergo enzymatic fermentation triggered by polyphenol oxidase and catalase resulted in structural variations in catechins thus formed theaflavin and thearubigins [1–3]. Structurally, theaflavin composed of catechins co-oxidation oriented benzotropolone skeleton coupled with dihydroxy and trihydroxy moiety at ortho- and vic-positions. Furthermore, oxidative modification of catechins and gallo-functional groups resulting in four major types of theaflavin along with some minor constituents [4]. Typically, theaflavin encompasses theaflavin (TF1), theaflavin-3-gallate (TF2A), theaflavin-3′gallate (TF2B) and theaflavin-3, 3′-digallate (TF3). Different catechins combinations are responsible for the diversity in theaflavin. Epicatechin combines with epigallocatechin and brings about TF1 while, EC and EGCG combine to form TF2A. Similarly, TF2B and TF3 are produced when ECG & EGC and ECG & EGCG bound to each other, respectively [5, 6]. Theaflavin is a natural antioxidant & metal chelator due to the presence of hydroxy groups however, the gallic acid moiety is also a contributory factor [7]. The majority of the studies have reported elevated antioxidant efficiency of theaflavin by esterification of hydroxyl ring with digallate esters [8]. Furthermore, it has been revealed that the inhibitory concentration (IC_50_) of theaflavin as a superoxide scavenger is higher than epigallocatechin gallate (EGCG). Likewise, preventive role of theaflavin in lipid peroxidation is mainly attributed to its ability to cease the chain reaction. Apart from free radical scavenging and metal chelating abilities, theaflavin has potential to activate certain antioxidant enzymes like glutathione-S-transferase (GST), glutathione peroxidase (GPX), superoxide dismutase (SOD) and catalase (CAT) thereby reduces lipid peroxidation. Likewise, thearubigins or polymeric black tea polyphenols (PBPs) are the oxidative products of phenolics and their production accelerated after 75% conversion of catechins into flavan-3-ol molecules. They have ability to activate phase II enzymes by inducing transcriptional upregulation in lung and liver [9].

Chronic renal failure (CRF) is an irreversible loss to functioning nephrons resulting numerous disorders of blood vessels, glomeruli, tubules and renal interstitium [10]. Various investigations have explicated the protective effect of black tea polyphenols against renal failure. The preventive role lies in their ability to reduce uremic toxins, nitric oxide production and enhance overall antioxidant status. They up regulate blood urea nitrogen and glamorous filtration alongside improve liver performance [11].

The mandate of current investigation to isolate the theafavins and thearubigins from black tea Qi-men variety by using different solvents and time intervals, in vitro characterization and preparation of nutraceutical intervention against renal malfunctioning in arginine induced liver damage rats.

## Methods

Black tea variety (Qi-Men) was procured from the National Tea Research Institute (NTRI), Shinkiari, Mansehra. The reagents (analytical and HPLC grade) and standards were purchased from Merck (Merck KGaA, Darmstadt, Germany) and Sigma-Aldrich (Sigma-Aldrich Tokyo, Japan). For efficacy trial, Male Sprague Dawley rats were housed in the Animal Room of Physiology department of GCUF. For biological assay, diagnostic kits were purchased from Sigma-Aldrich, Bioassay (Bioassays Chemical Co. Germany) and Cayman Chemicals (Cayman Europe, Estonia).

### Isolation of theaflavin and thearubigins

Theaflavin and thearubigins were extracted by using water, methanol and ethanol at 30, 60 and 90 min intervals and isolated by solvent partition method [12]. Initially, extracts except water based were concentrated through Rotary Evaporator (Eyela, Japan) and after filtration subjected to solvent partition using chloroform, ethyl acetate and butanol. Theaflavin and thearubigins rich fractions were separated followed by rotary evaporation and freeze drying (CHIRST, Alpha 1–4 LD plus, Germany).

### HPLC quantification

The isolated samples of theaflavins were characterized for their fractions through HPLC (PerkinElmer, Series 200, USA). The conditions for HPLC were C_18_ column (250 mm × 4.6 mm, 5.0 μm particle size), 10 μL sample through auto sampler (WISP Model 710) and column temperature 40 °C. The composition of mobile phase was acetonitrile, ethylacetate and 0.05% phosphoric acid in ratio of 21:3:76 with flow rate of 1 mL/min and using on U*V*/*v*is detector (model 481) and measurement wavelength was 278 nm.

### Indicators for lipid peroxidation

Antioxidant capacity of isolated theaflavin and thearubigins fractions were determined through antioxidant indices including total antioxidant activity, free radical scavenging activity and ferric reducing antioxidant power as following methods. For this purpose, the isolated fractions both from theaflavin and thearubigins were mixed in water, methanol and ethanol (1 mg/mL) to be further utilized in antioxidant indices estimation.

### Total polyphenols

Total phenolics of resultant isolates were estimated spectrophotometricaly using Folin-Ciocalteau method [13]. Briefly, equal amount of isolate in respective solvent and Folin- Ciocalteau reagent (125 μL) and distilled water (500 μL) were added and sty for 5 min at 22 °C. Afterwards, 4.5 mL of sodium bicarbonate solution (7%) was added to the mixture and absorbance was measured at 765 nm using a U*V*/*v*is Spectrophotometer (CECIL CE7200) against control and expressed results as mg gallic acid/100 g.

### Antioxidant activity

β-carotene and linoleic acid assay was applied to measure the total antioxidant capacity [14]. Purposely, a mixture of β-carotene, chloroform, linoleic acid and Tween20was mixed at ratio of 2 mg, 20 mL, 40 mg and 400 mg, respectively. Chloroform was removed and 3 mL of the remained emulsion with 0.10 mL sample put in a water bath for reaction for two hours. Oxidation of ß-carotene was measured at 470 nm by using spectrophotometer.

### Free radical scavenging activity (DPPH assay)

DPPH radical scavenging activity was measured according the procedure of [15]. Initially, DPPH (1 mL) was added to each extract (4 mL) and stay for 30 min on room temperature. The absorbance was noted at 520 nm using Spectrophotometer (CECIL CE7200).

### Ferric reducing antioxidant power (FRAP)

The FRAP test was performed according to the guidelines of [16]. 0.5 mL of respective extract was added in phosphate buffer (1.25 mL, 0.2 M, pH 6.6) and potassium ferricyanide (1.25 mL, 1%). After incubation, 10% TCA (1.25 mL) along with 0.1% ferric chloride were added in the mixture and then left at room temperature for 10 min and absorbance was measured at 700 nm.

### Glucose diffusion

Effect of isolated theaflavin and thearubigins fraction on glucose diffusion was assessed using glucose oxidase kit and 1.65 mM D-glucose solution [17].

### Experimental diet preparation

For biological assay, rats were divided in to four homogeneous groups fed on experimental diet. The common experimental diet was formulated using corn oil (10%), protein (10%), corn starch (64%), cellulose (10%), mineral (3%) vitamin mixture (1%) alongside arginine @ 2% for the induction of renal malfunctioning. For comparison a control study was also conducted by providing the normal diet (same composition except for arginine). However, common experimental diet from both studies further divided into four groups on the bases of addition of active ingredients, Diet 1 (Control, No active ingredient), Diet 2, (theaflavins supplementation @ 1 g), Diet 3, (Thearubigins supplementation @ 1 g) and Diet 4 (Supplementation of theaflavins+ thearubigins @ 0.5 + 0.5 g, respectively). Afterwards All the ingredients were mixed then oven baked for 10 min. The dose was selected by carried out a 21 days safety trial (Data not included).

In safety trial, rats were provided all the active ingredients orally @ of 250, 500, 1000, 1500, 2000 and 3000 mg/kg-bw (*n* = 6 rats/dose). The treatments were given in both acute (single dose followed by a 48-h observation period) and sub-acute (daily doses, except the highest, for 21 days). The hematological analysis as well as general physical examination was carried out [18].

### Study protocol

To evaluate the therapeutic potential of tested compounds against renal malfunctioning an efficacy trial was planned. For the purpose, 50 male Sprague Dawley rats were housed in the Animal Room of Institute of Home & Food Sciences, GCUF, Faisalabad. The protocol for this biological study was approved from Departmental ethical committee of GCUF that was in compliance with international standards ERC NO 2121. The rats were acclimatized by feeding on basal diet for a period of 1 week. The environmental conditions were control throughout the trial like temperature (23 ± 2 °C) and relative humidity (55 ± 5%) along with 12 h light-dark period. At the initiation of study, some rats (total 12 rats and average of results were considered as base line trend) were sacrificed to establish the baseline trend. For the induction of renal malfunctioning initially high arginine diet @ 2% was administrated for a period of 7 days. During that tenure urea and creatinine levels were observed to estimate the onset of renal malfunctioning. Afterwards when values of both test deviate 25% from normal then the original study was started. The study comprised of four groups of rats ten in each (Sample size according to power analysis). Accordingly, four types of experimental diets were given i.e. T0 (Basic experimental Diet), T1(Basic experimental Diet+ theaflavins supplementation @ 1 g/Kg B.W), T2 (Basic experimental Diet+ Thearubigins supplementation 1 g/Kg B.W) and T3 (Basic experimental Diet+ Supplementation of theaflavins+ thearubigins @ 0.5 + 0.5 g/ Kg B.W, respectively. During the 8 weeks trial, instantaneous administration of nutraceutical to experimental rats was ensured to assess their therapeutic role. At the termination of the study, overnight fasted rats were decapitated and blood was collected. For serum collection, blood samples were subjected to centrifugation using centrifuge machine @ 4000 rpm for 6 min. The respective sera samples were examined for various biochemical assays by using Microlab 300, Merck, Germany. Different biochemical parameters including serum urea and creatinine status, liver function test, antioxidant status, lipid profile alongside glucose & insulin and hematological analysis were accessed using respective commercial kits.

### Serum lipid profile

Serum cholesterol level was assessed by using CHOD–PAP method, likewise, low density lipoproteins (LDL) by protocols of [19], high density lipoprotein (HDL) by HDL Cholesterol Precipitant method [20] and triglycerides level through liquid triglycerides (GPO–PAP) method [19].

### Serum glucose and insulin levels

Glucose concentration was estimated by GOD-PAP method by following the protocol of [21], however, insulin level was determined by the method of [22].

### Kidney function test

The serum samples were also analyzed for urea by GLDH-method and creatinine by Jaffe-method using commercial kits [23] to assess the renal functionality of different groups.

### Antioxidant status

Glutathione contents were assessed by adapting the guidelines as mentioned by [24]. The reaction of product of GSH + DTNB in the protein free supernatant was estimated at 412 nm and expressed as nmol/mg protein. Similarly, Plasma Malondialdehyde (MDA) was also measured by estimation the colored product formation at 532 nm by the reaction between Lipid peroxidation generates peroxide intermediates MDA with Thiobarbitutic Acid (TBA) [25].

### Safety assessment

Liver function tests including aspartate aminotransferase (AST), alanine aminotransferase (ALT) and alkaline phosphatase (ALP) were assessed. Levels of AST and ALT were measured by the dinitrophenylhydrazene (DNPH) method using Sigma Kits 59–50 and 58–50, respectively and ALP by Alkaline Phosphates–DGKC method [26].

### Hematological aspects

Red blood cells indices including total red blood cells (TRBCs), hemoglobin (Hb), hematocrit (Hct) and mean corpuscular volume (MCV) were estimated. Likewise, white blood cell indices including monocytes, lymphocytes and neutrophils were measured by using Automatic Blood Analyzer (Nihon Kohden, Japan) [27].

### Statistical analysis

Statistical program SAS (version 9.1; Cary, NC) was utilized to analyze the data collected from this study. Two-way analysis of variance (ANOVA) was conducted to evaluate the effect of extraction time and solvent on polyphenol isolation. Moreover, One-way ANOVA were applied in efficacy trial and significance among the treatments were determined by applying LSD.

## Results

### Isolate characterization

#### Extraction yield

Means for extraction yield illuminated the significant effect of solvents (*p* ≥ 0.001) and time (*p* ≥ 0.002), highest theaflavin (3.42 ± 0.27 g/100 g) were detected in ethanolic extract followed by methanol (2.77 ± 0.25 g/100 g) whilst water exhibited the lowest yield (2.32 ± 0.13 g/100 g). Similarly, solvent (*p* ≥ 0.001) & time (*p* ≥ 0.001) also imparted pronounced impact for thearubigins, highest in ethanol and minimum in water. Extraction efficiency was also influenced by time and maximum yield for theaflavins and thearubigins was obtained at 60 min 3.41 ± 0.17 g/100 g and 13.17 ± 0.59 g/100 g, respectively (Table 1).

**Table 1:** Extraction yield of theaflavin and thearubigins

Solvents	Time			Means
	30 min	60 min	90 min	
Theaflavin (g/100 g black tea)				
Ethanol	3.11 ± 0.20	4.05 ± 0.19	3.12 ± 0.10	3.42 ± 0.27a
Methanol	2.05 ± 0.16	3.25 ± 0.15	3.01 ± 0.08	2.77 ± 0.25b
Water	1.99 ± 0.04	2.95 ± 0.19	2.02 ± 0.04	2.32 ± 0.13c
	2.38 ± 0.1c	3.41 ± 0.1a	2.71 ± 0.1b	
Thearubigins (g/100 g black tea)				
Ethanol	12.11 ± 0.20	15.32 ± 0.19	12.12 ± 0.10	13.18 ± 1.07a
Methanol	11.05 ± 0.16	13.25 ± 0.15	12.01 ± 0.08	12.10 ± 1.05b
Water	8.99 ± 0.04	10.95 ± 0.19	9.32 ± 0.04	9.75 ± 0.42c
	10.71 ± 0.4b	13.17 ± 0.5a	11.15 ± 0.9c	

Values are mean ± SD. Values in same column within each parameter with different letters were significantly different from each other (*p* ≤ 0. 05)

### HPLC quantification of individual fractions of theaflavins

**Fig. 1:**
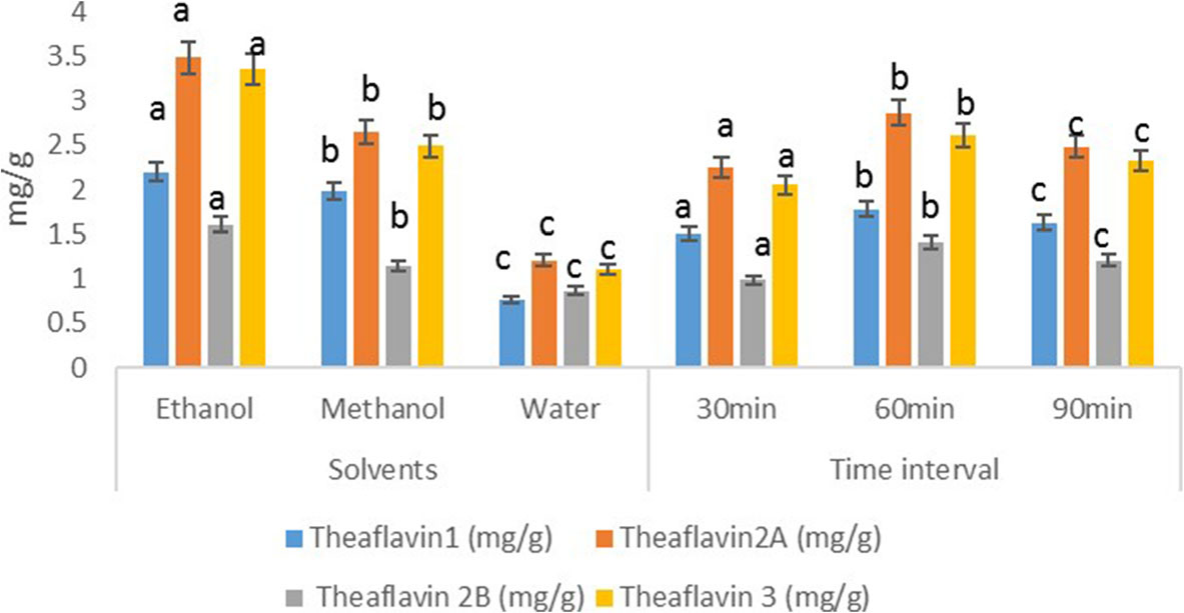
Absolute values for different isolated theaflavin fractions characterize through reverse phase HPLC in mg/g. Three solvents and time intervals were applied, and comparison were made between solvents and time intervals. Values with different letters were significantly different from each other (*p* ≤ 0. 05)

The theaflavins fractions were significantly affected by solvents (*p* ≥ 0.001) and extraction time (*p* ≥ 0.0031) whilst their interactive effect showed non-significant trend (*p* ≥ 0.521). Means for TF1 indicated highest value (2.18 ± 0.06 mg/g) in ethanolic extract followed by methanol (1.96 ± 0.03 mg/g) and water (0.751 ± 0.001 mg/g). However, effect of time revealed maximum output (1.79 ± 0.006 mg/g) at 60 min and minimum (1.48 ± 0.001 mg/g) at 30 min. Likewise trend was observed for TF2A, TF2B & TF3, among the solvents highest ratio in ethanolic fractions and minimum in water and among time intervals maximum in 60 min and minimum in 30 min (Fig. 1).

### In vitro antioxidant capacity (lipid peroxidation indicators)

Antioxidant indices of isolated theaflavins & thearubigins showed significant effect of solvents and time intervals for DPPH (*p* ≥ 0.001), ß-carotene (*p* ≥ 0.004) and FRAP (*p* ≥ 0.000) whilst non-significant differences (*p* ≥ 0.185) were observed for glucose diffusion. Means for DPPH in theaflavin and thearubigins indicated highest activity in ethanolic extract 83.27 ± 1.23 & 68.72 ± 3.61%, respectively followed by methanol and water. For the time factor, the highest DPPH activity of theaflavin was 82.69 ± 5.10% at 60 min whilst 78.03 ± 4.31 & 75.89 ± 3.92% at 90 and 30 min, respectively. Similarly, thearubigins also exhibited highest DPPH activity at 60 min (Table 2).

**Table 2:** Antioxidant indices of isolated theaflavin and thearubigins

Solvents	Time			Means
	30 min	60 min	90 min	
DPPH activity (theaflavin)%				
Ethanol	80.21 ± 2.31	87.51 ± 4.21	82.11 ± 5.91	83.27 ± 1.23a
Methanol	75.10 ± 2.60	81.08 ± 4.30	78.03 ± 3.60	78.08 ± 3.74b
Water	72.37 ± 3.12	79.49 ± 4.92	75.97 ± 4.92	75.94 ± 2.65c
	75.89 ± 3.92c	82.69 ± 5.10a	78.03 ± 4.31b	
DPPH activity (thearubigins)%				
Ethanol	62.58 ± 3.21	74.26 ± 1.90	69.33 ± 4.01	68.72 ± 3.61a
Methanol	60.75 ± 3.26	63.26 ± 1.42	61.03 ± 1.36	61.68 ± 2.69b
Water	58.18 ± 4.32	62.03 ± 1.93	60.13 ± 3.02	60.11 ± 1.36b
	60.50 ± 2.36c	66.51 ± 1.32a	63.49 ± 2.13b	
ß-carotene (theaflavin)%				
Ethanol	63.16 ± 2.31c	73.23 ± 3.60a	69.85 ± 3.42b	68.74 ± 4.31a
Methanol	60.15 ± 3.62e	67.35 ± 2.91d	65.12 ± 4.13d	64.20 ± 4.72b
Water	59.61 ± 4.12 h	64.16 ± 3.12 g	61.04 ± 1.94f	61.27 ± 2.30c
	62.61 ± 3.12c	68.91 ± 2.42a	65.33 ± 4.22b	
ß-carotene (thearubigins)%				
Ethanol	60.16 ± 2.31c	70.23 ± 2.81a	66.85 ± 3.10b	63.74 ± 1.22a
Methanol	58.15 ± 1.52e	68.35 ± 2.33d	62.12 ± 4.31d	62.87 ± 3.20b
Water	56.61 ± 2.90 h	65.16 ± 1.92 g	61.04 ± 3.62f	60.93 ± 4.16b
	59.30 ± 3.13c	67.91 ± 2.31a	63.33 ± 4.23b	

Values are mean ± SD. Values in same column within each parameter with different letters were significantly different from each other (*p* ≤ 0. 05)

Likewise, highest ß-carotene activity of theaflavin and thearubigins were observed in ethanolic extract and at 60 min time interval. Likewise, The FRAP values for theaflavin and thearubigins were detected highest in ethanolic extract and at 60 min. The highest glucose diffusion was noticed in ethanolic extract of theaflavin (91.74 ± 4.16%) followed by the methanolic (89.96 ± 2.31%) and water extract (87.02 ± 3.12%). Likewise, in thearubigins the values for this parameter in respective extracts were 87.71 ± 6.12, 84.92 ± 6.42 and 83.02 ± 7.12%. Considering time factor, highest glucose diffusion was reported at 60 min for the both parameters (Table 3) .

**Table 3:** FRAP and Glucose diffusion of isolated theaflavin and thearubigins

Solvents	Time			Means
	30 min	60 min	90 min	
FRAP (theaflavin) μmol Fe2^+^/g				
Ethanol	811.67 ± 10.21	958.33 ± 8.48	799.33 ± 12.31	856.44 ± 9.06a
Methanol	698.67 ± 10.36	704.00 ± 8.65	658.67 ± 11.32	687.11 ± 12.03b
Water	489.67 ± 10.56	612.25 ± 8.23	586.67 ± 13.01	562.86 ± 11.01c
	666.67 ± 10.54c	758.19 ± 8.21a	681.55 ± 9.06b	
FRAP (thearubigins) μmol Fe2^+^/g				
Ethanol	690.02 ± 1.23	845.01 ± 7.45	701.33 ± 12.23	745.44 ± 8.21a
Methanol	538.67 ± 1.56	665.00 ± 7.49	591.67 ± 11.32	598.44 ± 11.20b
Water	429.67 ± 10.23	537.33 ± 7.52	503.67 ± 10.25	490.22 ± 12.20c
	552.70 ± 10.21c	682.40 ± 12.23a	598.89 ± 9.12b	
Glucose diffusion (theaflavin)%				
Ethanol	90.37 ± 3.60	95.99 ± 6.34	90.88 ± 4.13	91.74 ± 4.16
Methanol	89.87 ± 4.21	93.69 ± 4.21	89.22 ± 6.12	89.96 ± 2.31
Water	87.30 ± 2.62	90.36 ± 5.32	88.40 ± 3.21	87.02 ± 3.12
	89.18 ± 5.20	93.34 ± 3.12	89.50 ± 4.13	
Glucose diffusion (thearubigins)%				
Ethanol	85.27 ± 2.3	89.99 ± 2.9	87.88 ± 4.2	87.71 ± 6.12
Methanol	83.87 ± 1.3	87.69 ± 3.2	83.22 ± 3.6	84.92 ± 6.42
Water	81.30 ± 3.6	85.36 ± 2.9	82.40 ± 4.2	83.02 ± 7.12
	83.48 ± 4.32	87.68 ± 4.13	84.50 ± 6.34	

Values are mean ± SD. Values in same column within each parameter with different letters were significantly different from each other (*p* ≤ 0. 05)

### Biological assay

#### Weight gain

The Mean values regarding body weights of experiments rats showed significant effect (*p* ≥ 0.001) of experimental diets on weight in both control and renal malfunctional rats and recorded values for weight in renal malfunctional rats at the termination of study were 213.10 ± 6.11 & 213.73 ± 8.12, 217.74 ± 5.61 & 220.52 ± 9.26 and 214.17 ± 4.25 & 215.73 ± 6.23 g/rat for T_1_ T_2_ and T_3_, respectively (Table 4).

**Table 4:** Effect of theaflavin and thearubigins and their combination based intervention on selected traits of normal and renal malfunctional rats at the termination of the study (56th day)

Parameters	Base line values (*n* = 1)	Treatments	Post treatment values at termination of study Control Study (Normal Rats)	Post treatment values at termination of study Arginine induced renal malfunctional rats
Body weight (at the termination of study)	128 ± 10.25	T0	224.18 ± 10.25a	223 ± 9.61a
		T1	212.14 ± 9.23d	213.10 ± 6.11c
		T2	216.41 ± 8.56b	217.74 ± 5.61b
		T3	213.12 ± 9.22c	214.17 ± 4.25c
TC (mg/dL)	77.80 ± 3.56	T0	85.14 ± 4.12a	86.31 ± 4.63a
		T1	82.13 ± 3.25c	81.14 ± 2.12c
		T2	84.15 ± 2.212b	82.99 ± 5.63b
		T3	83.1 ± 3.14c	82.29 ± 4.25b
LDL-C (mg/dL)	26.56 ± 3.56	T0	27.10 ± 0.96a	33.36 ± 2.13a
		T1	26.01 ± 1.01c	30.94 ± 1.22c
		T2	26.65 ± 0.95b	32.20 ± 3.61b
		T3	26.30 ± 1.02b	31.49 ± 2.81b
HDL-C (mg/dL)	32.56 ± 3.56	T0	38.45 ± 1.1	38.54 ± 2.36
		T1	39.21 ± 0.96	39.70 ± 2.32
		T2	39.01 ± 0.85	38.92 ± 1.09
		T3	39.14 ± 0.89	39.64 ± 1.56
TG (mg/dL)	58.26 ± 3.56		69.45 ± 1.20a	67.15 ± 4.20
		T1	67.12 ± 1.14c	64.96 ± 5.15
		T2	68.01 ± 1.05b	65.20 ± 4.12
		T3	67.65 ± 1.08c	65.13 ± 2.12
Glucose (mg/dL)	79.63 ± 3.72	T0	85.65 ± 2.2a	89.18 ± 6.14a
		T1	83.15 ± 1.96b	84.70 ± 6.13b
		T2	83.69 ± 1.12b	85.60 ± 3.20b
		T3	82.38 ± 1.23c	83.98 ± 7.14b
Insulin μU/Ml	6.23 ± 0.56	T0	8.25 ± 0.01b	7.76 ± 0.22b
		T1	8.91 ± 0.25a	8.06 ± 0.41a
		T2	8.80 ± 0.31b	7.96 ± 0.52ab
		T3	8.99 ± 0.21a	8.11 ± 0.12a
Serum Urea mg/Dl	32.56 ± 1.03	T0	26.14 ± 1.01a	37.83 ± 2.46a
		T1	25.96 ± 1.04b	34.09 ± 2.56b
		T2	26.01 ± 0.96a	34.95 ± 2.91b
		T3	25.78 ± 0.88b	33.44 ± 1.45c
Serum creatinine (mg/dL)	1.01 ± 0.02	T0	0.719 ± 0.01	1.15 ± 0.03a
		T1	0.715 ± 0.02	1.08 ± 0.01b
		T2	0.716 ± 0.01	1.09 ± 0.021b
		T3	0.701 ± 0.03	1.04 ± 0.05c
Serum glutathione (mg/L)	42.32 ± 1.02	T0	50.52 ± 1.01b	43.12 ± 3.01c
		T1	52.99 ± 1.45ab	46.19 ± 2.56b
		T2	52.14 ± 1.21ab	44.91 ± 4.14bc
		T3	53.01 ± 1.24a	47.87 ± 4.12a
Malonialdehyde (MDA) (mmol/L)	6.23 ± 0.59	T0	7.85 ± 0.14a	8.01 ± 0.21a
		T1	6.23 ± 0.18b	6.78 ± 0.24c
		T2	7.15 ± 0.12a	7.55 ± 0.01a
		T3	6.85 ± 0.13ab	6.95 ± 0.42b
AST (IU/L)	6.23 ± 0.59	T0	7.85 ± 0.14a	8.01 ± 0.21a
		T1	6.23 ± 0.18b	6.78 ± 0.24c
		T2	7.15 ± 0.12a	7.55 ± 0.01a
		T3	6.85 ± 0.13ab	6.95 ± 0.42b
ALT(IU/L)	6.23 ± 0.59	T0	7.85 ± 0.14a	8.01 ± 0.21a
		T1	6.23 ± 0.18b	6.78 ± 0.24c
		T2	7.15 ± 0.12a	7.55 ± 0.01a
		T3	6.85 ± 0.13ab	6.95 ± 0.42b
ALP(IU/L)	6.23 ± 0.59	T0	7.85 ± 0.14a	8.01 ± 0.21a
		T1	6.23 ± 0.18b	6.78 ± 0.24c
		T2	7.15 ± 0.12a	7.55 ± 0.01a
		T3	6.85 ± 0.13ab	6.95 ± 0.42b

T0: Group rely on Basic Experimental Diet

T1: Group rely on Basic Experimental Diet+ theaflavins supplementation @ 1 g

T2: Group rely on Basic Experimental Diet+ Thearubigins supplementation @ 1 g

T3: Group rely on Basic Experimental Diet+ Supplementation of theaflavins+ thearubigins @ 0.5 + 0.5 g, respectively

Values are mean ± SEM (*n* = 10)

One way anova was applied to check the overall behavior of the study parameter to elaborate the effect of treatments on selected parameter of rats at the termination of study. To evaluate the differences among the mean LSD test was applied. Values in same column within each parameter with different letters were significantly different from each other (*p* ≤ 0. 05)

### Lipid profile

The prepared diets caused significant (*p* ≥ 0.000) effect on serum cholesterol level of rats, highest cholesterol level was observed in T_0_ (86.31 ± 4.63 mg/dL) that significantly reduced in T_1_ (81.14 ± 2.12 mg/dL) trailed by T_3_ (82.29 ± 4.25 mg/dL) and T_2_ (82.99 ± 5.63 mg/dL). In contrary, the tested diets imparted non-significant (*p* ≥ 0.091) enhancement in HDL levels of study animals. In this context, T_0_ drink consuming group exhibited least HDL value as 38.54 ± 2.36 mg/dL, respectively that elevated non-significantly to 39.70 ± 2.32, 38.92 ± 1.09 and 39.64 ± 1.56 mg/dL in T_1,_ T_2_ and T_3_ groups_,_ respectively. As for as LDL levels were concern, the tested diets caused momentous (*p* ≥ 0.000) decline on this trait. The maximum LDL was noticed in T_0_ that reduced substantially to in T_1_, T_2_ and T_3,_ correspondingly_._ Triglycerides values for T_0_, T_1_, T_2_ and T_3_ differed non-momentously (*p* ≥ 0.128) i.e. 67.15 ± 4.20, 64.96 ± 5.15, 65.20 ± 4.12 and 65.13 ± 2.12 mg/dL (Table 4). Likewise trend was observed for normal experimental rats, all the experimental diets caused significant impact on total cholesterol and LDL as compared to control.

### Glucose & Insulin Levels

Mean values showed significant (*p* ≥ 0.000) impact of experimental diets on blood glucose levels of tested groups. In renal malfunctional rats, the highest glucose level 89.18 ± 6.14 in T_0_ group however, black tea polyphenols supplemented diets lowered the glucose value to 84.70 ± 6.13, 85.60 ± 3.20 and 83.98 ± 7.14 mg/dL in T_1,_ T_2_ and T_3_ groups, correspondingly. Likewise, insulin also varied significantly (*p* ≥ 0.004) minimum insulin values 7.76 ± 0.22 μU/mL were observed in T_0_ whilst highest level 8.11 ± 0.12 μU/mL in T_3_ followed by 8.06 ± 0.41 μU/mL in T_1_ and 7.96 ± 0.52 μU/mL in T_2_ (Table 4).

### Serum Urea & Creatinine levels

The means in Table 4 regarding the serum urea & creatinine levels reveled that theaflavin and thearubigins supplemented diets divulged significant (*p* ≥ 0.001 & *p* ≥ 0.000, respectively) impact on these traits however, the diet supplemented with both theaflavin and thearubigins caused maximum effect in comparison with others. The highest urea level 37.83 ± 2.46 was recorded in T_0_ that suppressed to 33.44 ± 1.45, 34.09 ± 2.56 and 34.95 ± 2.91 mg/dL in T_3,_ T_1_ and T_2_ groups. Likewise, highest creatinine level 1.15 ± 0.03 mg/dL was recorded in T_0_ group (control drink) that significantly suppressed to 1.04 ± 0.05 mg/dL in T_3_ (drink containing theaflavin+thearubigins), 1.08 ± 0.01 mg/dL in T_1_ (drink containing theaflavin) and 1.09 ± 0.02 mg/dL in T_2_ (drink containing thearubigins).

### Antioxidant status

T_0_ group showed decreased glutathione content 43.12 ± 3.01 mg/L that momentously (*p* ≥ 0.001) enhanced to 46.19 ± 2.56, 44.91 ± 4.14 and 47.87 ± 4.12 mg/L in T_1,_ T_2_ and T_3_ groups. Similar significant (*p* ≥ 0.000) effect of experimental diets was observed for MDA, the recorded values for this trait in T0 was 8.01 ± 0.21that differed significantly in T1, T2 and T3 by 6.78 ± 0.24, 7.55 ± 0.01 and 6.95 ± 0.4 mmol/L (Table 4). Again, the more pronounced effect was observed for T3 (drink containing theaflavin+thearubigins) followed by T1 (drink containing theaflavin) & T2 (drink containing thearubigins).

### Liver function tests

Mean values in Table 4 showed pronounced effect of experimental diets on liver function enzymes. In this scenario, ALT value in T_0_ (49.06 ± 3.01 IU/L) was significantly ((*p* ≥ 0.001)) varied in T_1_ T_2_ and T_3_ groups with mean values 48.50 ± 2.42, 49.12 ± 2.13 and 46.38 ± 2.45 IU/L. Moreover, T_0_ group showed maximum AST level 105.27 ± 5.14 IU/Lthat reduced substantially ((*p* ≥ 0.002) in T_1_ (101.68 ± 9.12 IU/L), T_2_ (103.00 ± 9.02 IU/L) and T_3_ (109.01 ± 9.45 IU/L). Likewise, ALP level in T_0_ (196.89 ± 17.23 IU/L) was significantly ((*p* ≥ 0.001)) higher than that of T_1_ (177.89 ± 15.20 IU/L), T_2_ (180.90 ± 13.25 IU/L) and T_3_ (167.68 ± 9.62 IU/L).

### Hematological analysis

**Fig. 2:**
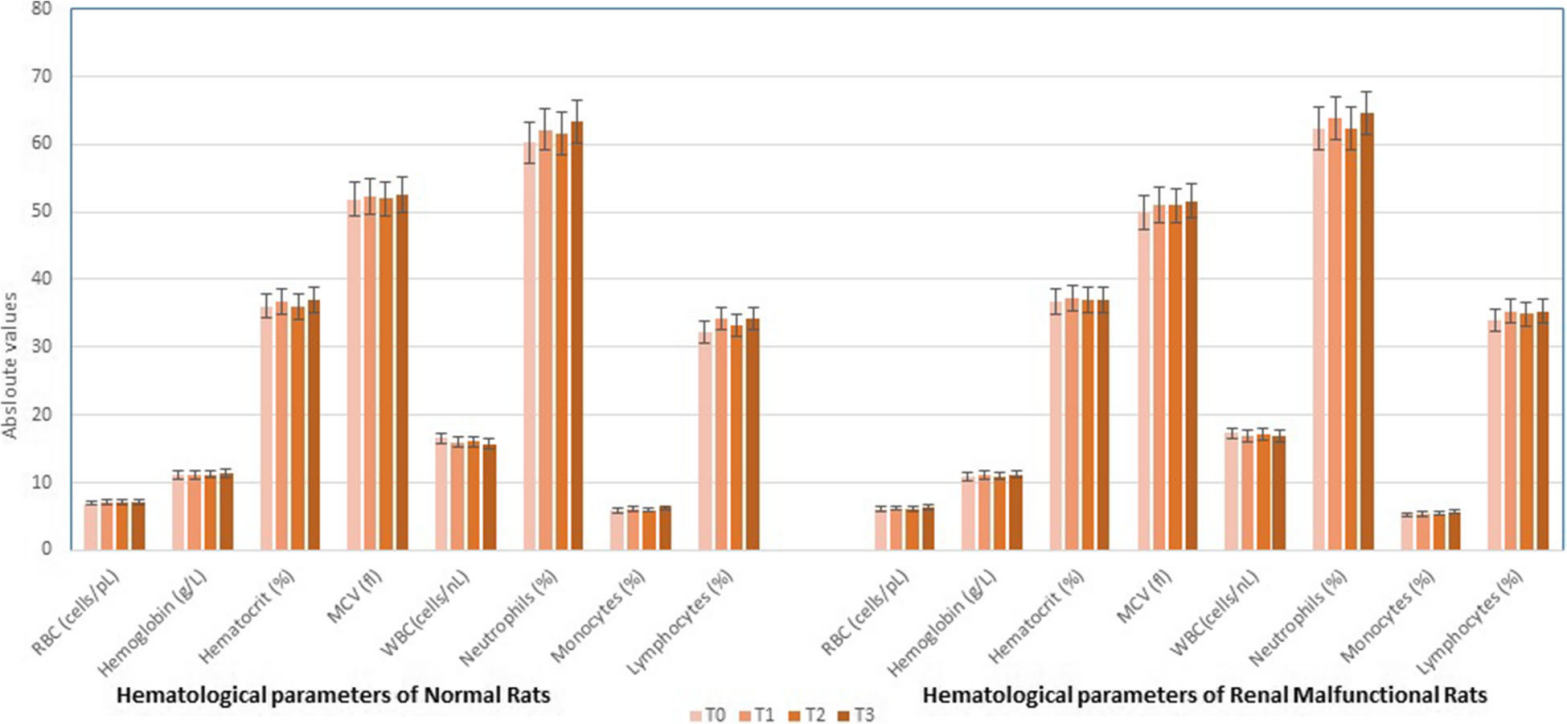
Absolute values for Hematological indices (Red blood cell indices and White cell indices) of normal & arginine induced renal malfunction rats at the termination of study (56th days). The experimental diets T0 (Group rely on Basic Experimental Diet), T1 (Group rely on Basic Experimental Diet+ theaflavins supplementation @ 1 g) T2 (Group rely on Basic Experimental Diet+ Thearubigins supplementation @ 1 g) & T3 (Group rely on Basic Experimental Diet+ Supplementation of theaflavins+ thearubigins @ 0.5 + 0.5 g, respectively) were given through entire study period. Values are mean ± SEM (*n* = 10) and level of significance were determined at (*p* ≤ 0. 05)

Mean values for heamatological indices indicated non-significant differences due to treatments on red and white cell indices. The recorded RBCs values were 6.13 ± 0.22, 6.21 ± 0.82, 6.15 ± 0.25 and 6.39 ± 0.63 cells/pL in T_0,_ T_1,_ T_2_ and T_3,_ respectively. Mean Hb level in T_0,_ T_1,_ T_2_ and T_3_ were 10.88 ± 0.71, 11.15 ± 0.61, 10.98 ± 0.81 and 11.20 ± 0.71 g/L_,_ respectively. Moreover, the recorded hematocrit value for T_0_ (36.79 ± 1.52%) was improved non-significantly in T_1_, T_2_ and T_3_ groups as 37.25 ± 2.71, 36.99 ± 2.12 and 37.03 ± 2.81%, respectively. Likewise, mean MCV values for T_0_, T_1,_ T_2_ and T_3_ were 49.96 ± 2.48, 51.03 ± 3.82, 50.92 ± 3.97 and 51.65 ± 3.30 fl, respectively. Similarly, the mean WBCs in T_0_ were 17.29 ± 0.58 cells/nL that non-significantly decreased to 16.99 ± 0.49, 17.26 ± 0.65 and 16.85 ± 0.35 cells/nL, respectively in tested groups. Likewise, means for Neutrophils in T_0,_ T_1,_ T_2,_ and T_3_ were 62.25 ± 1.28, 63.85 ± 2.82, 62.45 ± 3.25 and 64.63 ± 2.63%, respectively. Mean monocytes values for T_0_, T_1,_ T_2_ and T_3_ groups were 5.29 ± 0.21, 5.35 ± 0.22, 5.41 ± 0.25 and 5.65 ± 0.63%, respectively. In study IV, values for **Lymphocytes** were 33.92 ± 1.21, 35.25 ± 1.82, 34.91 ± 1.25 and 35.29 ± 1.63% in T_0,_ T_1,_ T_2_ and T_3_ group_,_ respectively (Fig. 2).

## Discussion

In the current study we first time examined the synergistic role of isolated theaflavins and thearubigins against the indicators for lipid peroxidation both in in vitro and in vivo by utilizing excessive arginine induced renal malfunctional rats. The hall mark of the research is the optimization of these bioactive moieties isolation through different solvents and time intervals. The outcomes of study indicated that the isolation was affected by nature of solvent and intervals of extraction. The results clearly depicted that the extraction yield was associated with type of solvents and extraction time. In the current study, the significant effect of solvents on polyphenols recovery are also supported by the work of [28, 29], observed method of extraction and time of extraction are the considerate factors in this context. Extraction time and yield have a linear association however, nature of the compound is a detrimental factor for optimum recovery. The lower isolate recovery at elevated extraction time beyond 60 min might be due to thermal degradation. The high temperature may trigger the early decomposition of polyphenols resulting reduction in optimum recovery. Nevertheless, extraction at lower temperature required more time for inclusive isolation thereby exerting less harm to the polyphenols.

The antioxidant indices are the good indicators for compounds ability to provide protection against lipid peroxidation and also provide evidence for its in vivo therapeutic potential. The higher DPPH activity of theaflavins as compared to thearubigins is might be due to benzotropolone skeleton formation during co-oxidation of catechins, one with a vic-trihydroxy moiety and the other with an ortho-dihydroxy structure. Moreover, chemical structure of theaflavin contains two A-rings of flavanols linked by fused seven-member ring. These structural features provide more interaction sites to free radicals. Higher free radical and hydrogen peroxide quenching capacity of theaflavin is might be due to benzotropolone ring however, the ability of individual theaflavin moiety depends upon its gallate structure [30]. The lower DPPH activity of thearubigins as compared to theaflavins is due to the presence of structural differences between theaflavin and thearubigins. The theaflavin holds more galloyl moieties along with hydroxyl groups thus showed higher anti-radical ability [31]. Alongside, theaflavin also exhibited strong FRAP value owing to its ability to quench the generated Fe (II) free radical.

The higher amount of TF1, TF2, TF2B and TF3 were detected differently in different solvents. The difference is might be as a function of polarity of the solvent and nature of the theaflavin fractions. Moreover, individual theaflavins fraction in tea was dependent upon the plucking season, varietals variations and processing method along with analytical technique [32].

In the current exploration, more potent effect of theaflavin as lipid management drug as compared to its counterparts might be explicated through its ability to diminish the intestinal lipid absorption and pancreatic lipase activity [33]. Fatty acid synthase (FAS) enzyme imbalance is responsible for the initiation of different metabolic related abnormalities and FAS inhibitors can play a pivotal role. Accordingly, theaflavin manage the inhibition of FAS through downregulation of PI-3 K/AKT/ Sp-1 pathway [34]. Black tea polyphenols manage the lipid related abnormalities through different mechanistic routes like accelerating the fecal excretion of fatty acids and sterols, by modulating the satiety, thermogenesis and fat oxidation, stimulating the cellular energy expenditure and interfere with cholesterol micelle solubilization [35]. An array of evidences has proven the ability of black tea polyphenols especially theaflavin to reverse LDL oxidation by scavenging the free radicals, hindering the foam cell formation and deposition Antioxidative action is not solely the route by which tea inhibits LDL level in the subjects; tea polyphenols also have the ability to address this menace with some other mechanistic approaches. One of the key targets is the modulation of adiponectin level [36, 37]. Hypoglycemic ability of black tea is associated with their impact on the modulation of glucose carriers like GLUTs, translocation proteins IRβ, AMPKR and GLUT4 expressions that involved in the maintenance of glucose homeostasis. Oxidative stress induced reduction in the expression of GLUT4 and associated proteins resulting reduction in glucose incorporation in the cells thus amplified the glycemic abnormalities [38, 39]. Among the other mechanistic concerns improvement in the muscle glucose transporters, adipocytes and leptin reductions are the possible mechanisms by which black tea polyphenols perform their action for insulin management [40]. There are established evidences highlighted the therapeutic role of black tea polyphenols against kidney malfunctioning [12]. The continuous ingestion of black tea for a period of 8 weeks caused momentous reduction in blood urea owing to its high antioxidative potential. In a study [41], noticed elevation of urea and creatinine levels in rats fed on high arginine diet due to the production of uremic acid toxins and suppression of certain key hormones. The phenomenon of oxidative stress induced undesirable changes in kidney functionality that can be tackled by black tea polyphenols especially through theaflavin and thearubigins by quenching free radicals. The tea polyphenols modulate the arachidonic acid metabolism, suppressing the prostaglandin (PG), thromboxane A2 and cyclooxygenase expressions of arachidonic acid in microsomes and glomeruli thus helpful in the management of kidney abnormalities [42, 43]. They inferred that during oxidative stress some morphological abnormalities in glomerulus, capillaries and tubules are produced that can successfully be uplifted by black tea. Earlier [44], explicated that black tea modulates c-reactive proteins and uric acid production that ultimately reduce toxins from kidney, alleviate free radicals and inflammation. It is also worth mentioning that tea polyphenols resulted marked decline in the creatinine level by their action on platelets thus enables kidneys to regain their normal functioning. Moreover, the diuretic effect of black tea enhances renal blood flow, capillary expansion and glomerular filtration. They proposed that improvement in inflammation, sore lesion and deformation in tubules are the leading routes for renal modulating action. The inverse association between glutathione and oxidative stress has been unveiled in many research explorations. There are consolidated evidences in favor of black tea polyphenols capacity to combat oxidative stress by enhancing the activity of glutathione and other antioxidant enzymes. Moreover, it provides assistance through theaflavin and thearubigins to quench the free radicals (hydroxyl and superoxide anions) as a consequence of abnormal oxygen balance in the body. Theaflavin and thearubigins are the proven antioxidant and metal chelators that equally effective in vivo and in vitro models due to their instinct to trap the noxious radicals like superoxide and peroxyl [45]. Additionally, theaflavin and thearubigins assist to quench the hydroxyl radical, ferryl and superoxide radical anion. The majority of the studies elucidated that apart from free radical scavenging and metal chelating abilities. They have potential to activate certain antioxidant enzymes like glutathione-S-transferase (GST), glutathione peroxidase (GPX), superoxide dismutase (SOD) and catalase (CAT) thereby reduce lipid peroxidation. Previous studies showed that black tea polyphenols especially theaflavin have ability to attenuate the process of lipid peroxidation. Structurally, theaflavin is composed of vicinal dihydroxy and trihydroxy components and benzotropolone skeleton responsible for free radicals quenching and metal ions chelating potency. The altered hematological markers are the indicators of adverse effect of a diet or drug that ultimately cause multi-dimensional malfunctioning in the body. The abnormalities in both white and red blood cells indices are reported by different scientists during diseases state. They deduced that enhancement in microvescicles formation, production of excessive toxins and membrane oxidations are the possible reasons that induce elevation in white blood cells and decline in hemoglobin, red blood cells and monocytes [46]. In current study, theaflavin and thearubigins administration caused non-significant improvements in abnormal hematological parameters might be due to their antioxidative potential that helpful against oxidative stress.

## Conclusions

It is envisaged from present discussion that theaflavin and thearubigins based dietary intervention has proved effectual to assuage the oxidative stress related maladies. Moreover, extraction conditions also have impact on the yield of these bioactive moieties. Likewise, they imparted significant impact on the indices of lipid peroxidation both in vitro and in vivo thus suitable to tackle oxidative abnormalities. Moreover, it is recommended that in future more sophisticated methods like GS-NEM conjugates by HPLC or GSH recycling method should be adapted for the Gluthathione (GSH) determination to enhance the authenticity of the results. Likewise, human efficacy trial for insulin sensitivity/resistance by different test like HOMA-insulin resistance, QUIKI, and Matsuda should be conducted to unveil the mechanistic concerns.

## Abbreviations

ALP: Alkaline phosphatase
ALT: Alanine transaminase
AMPK: Activation of activated protein kinase
AST: Aspartate aminotransferase
CKD: Chronic kidney disease
CYP1A1: CYTOCHROME P450, SUBFAMILY I, POLYPEPTIDE 1
FAS: Fatty acid synthase enzyme
GLTU: Glucose transporters
GPX: Glutathione peroxidase
GSH-Px: Glutathione peroxidase
GST: Glutathione-S-transferase
HDL: High density Lipoprotein
IRβ: Insulin receptor β subunit
LDL: Low density Lipoprotein
MDA: Malondialdehyde
OHdG: 8-hydroxy-2′ -deoxyguanosine
PG: Prostaglandin
POD: Peroxidase
PPO: Polyphenoloxidase
ROS: Reactive oxygen spices
TC: Total Cholesterol
TFs: Theaflavins
TG: Triglycerides
TRBs: Thearubigins
VLDL: Very low density Lipoprotein
